# Minimal Residual Disease in Oncology: From Cure to Longitudinal Patient Management

**DOI:** 10.3390/cancers18071049

**Published:** 2026-03-24

**Authors:** Jinhee Kim, Franck Morceau, Yong-Jun Kwon, Yong Jae Shin

**Affiliations:** 1 Research Institute for Future Medicine, Samsung Medical Center, Seoul 06351, Republic of Korea; 2 Precision Medicine Technology, Translational Medicine Operations Hub, Luxembourg Institute of Health, 3555 Dudelange, Luxembourg; 3 School of Medicine, Sungkyunkwan University, Seoul 06351, Republic of Korea

**Keywords:** patient-informed minimal residual disease, circulating tumor DNA, predictive biomarkers, precision oncology

## Abstract

Minimal residual disease (MRD) refers to the persistence of low-level malignant cells or tumor-derived nucleic acids that remain after curative-intent therapy and are undetectable by conventional diagnostic methods. In oncology, MRD has emerged as a powerful biomarker with well-established prognostic value in hematologic malignancies and rapidly expanding relevance in solid tumors. Advances in sensitive detection technologies, including multiparameter flow cytometry, quantitative real-time polymerase chain reaction, next-generation sequencing, and digital polymerase chain reaction, have enabled the identification of residual disease at the molecular level, often preceding clinical or radiological relapse. Beyond its conventional role as a binary indicator of treatment response or cure, MRD is increasingly recognized as a dynamic longitudinal biomarker that supports personalized disease management. Within this evolving paradigm, patient-informed MRD strategies that incorporate tumor-specific molecular profiling and serial monitoring, particularly through circulating tumor DNA, offer the potential to guide treatment adaptation, including escalation, de-escalation, maintenance optimization, and surveillance strategies across both hematologic and solid malignancies. In this review, we summarize the biological basis of MRD, current and emerging detection methodologies, and clinical applications across cancer types, with a focus on patient-informed approaches. We also discuss key limitations, including assay standardization, biological variability in solid tumors, and the lack of clearly defined actionability thresholds. Finally, we highlight future directions for integrating MRD with multi-omics and AI-driven analytical frameworks to enable adaptive, risk-informed cancer management and advanced precision oncology.

## Simple Summary

Minimal residual disease (MRD) refers to very small numbers of cancer cells that are detectable in the body after curative treatment. Circulating tumor DNA (ctDNA) is a reliable biomarker for detecting MRD and predicting recurrence in hematologic malignancies and solid tumors using liquid biopsy techniques. However, MRD testing has certain limitations, including variable assay accuracy and lack of standardization across laboratories. Future advances in highly sensitive molecular methods, multi-omics analysis, and artificial intelligence are expected to improve reliability and to enable the continuous monitoring of cancer over time. Ultimately, the application of MRD has been shifting from curing cancer to personalized longitudinal disease management, but large clinical trials and standardized guidelines are needed before MRD can be universally applied in routine practice.

## 1. Introduction

Cancer is a complex disease characterized by the uncontrolled growth and spread of abnormal cells to other parts of the body, as defined by the National Cancer Institute (NCI) [1]. Cancers are typically classified according to their tissue site of origin. Carcinomas, which arise from epithelial cells, account for approximately 80–90% of human cancers and are subdivided into adenocarcinoma, occurring in the mucus membranes of organs or glands, and squamous cell carcinoma, originating from the squamous epithelium. Sarcomas are solid tumors derived from connective tissues, including muscle, fat, bones, tendons, and cartilage. Leukemias, also known as blood cancers, originate in the bone marrow (BM), the site of blood cell production, and typically develop through the overgrowth of immature white blood cells. Lymphomas arise from the lymphatic system, a network of vessels, nodes, and organs such as the spleen, tonsil, and thymus [2].

Cancer treatment strategies are multifaceted and include surgery to remove the entire tumor; chemotherapy to target rapidly dividing cells; targeted therapy to inhibit specific molecular pathways; hormone therapy for hormone-sensitive cancers; and immunotherapy to enhance the immune response against cancer [3].

The accurate diagnosis of cancer relies on a variety of techniques, including imaging, laboratory tests, endoscopy, surgery, genetic testing, and biopsy. Among these procedures, biopsy is an important tool and may be performed using needle, endoscopic, surgical, skin, or liquid approaches. Liquid biopsy is a minimally invasive sampling method that detects cancer-related components—such as circulating tumor cells (CTCs), circulating tumor DNA (ctDNA), tumor-derived extracellular vesicles (EVs), tumor-educated platelets, and circulating free RNA—in the blood or other body fluids [4,5]. Compared with tissue biopsy, liquid biopsy is particularly valuable for early screening. It plays a significant role in early cancer detection and in monitoring minimal residual disease (MRD) after treatment, thereby enabling personalized and timely therapeutic decisions [6,7]. MRD refers to the presence of a very small population of residual malignant cells or tumor-derived nucleic acids that persist after primary therapy. These residual cells may remain dormant, develop treatment resistance, or reside in sanctuary sites such as the BM or lymph nodes [8,9]. Owing to their extremely low abundance, MRD is typically undetectable using conventional imaging or standard pathological methods.

Advances in highly sensitive molecular technologies have substantially improved the ability to detect of MRD. In particular, circulating tumor DNA (ctDNA) analysis using liquid biopsy enables non-invasive monitoring of tumor-specific mutations in the bloodstream. These approaches allow clinicians to detect molecular relapse months before radiologic evidence of disease recurrence becomes apparent [6,7].

Early identification of MRD provides valuable prognostic information and may enable timely therapeutic intervention, ultimately improving long-term clinical outcomes [8,10].

Notably, MRD is no longer viewed solely as a binary marker distinguishing cure from relapse. Instead, it is increasingly recognized as a dynamic, longitudinal biomarker that supports personalized disease monitoring and treatment management. Within this evolving paradigm, MRD informs therapeutic escalation or de-escalation, maintenance duration, surveillance intensity, and treatment-free remission strategies in selected hematological settings [8,9,11]. This conceptual shift—from MRD as a marker of cure to a tool for ongoing disease management—represents a central advancement in precision oncology.

In this review, we discuss the biological basis of MRD and its relationship with metastasis, summarize established and emerging technologies for MRD detection, and evaluate their clinical utility across hematologic and solid malignancies. Particular emphasis is placed on patient-informed MRD strategies that integrate tumor-specific molecular profiling, longitudinal sampling, and advanced multi-omics approaches, while outlining current limitations and future directions for MRD-guided cancer care.

## 2. MRD and Metastasis

During MRD, surviving tumor cells may undergo dynamic genetic or epigenetic changes that enhance their ability to survive, evade the immune system, and metastasize. Some of these cells enter the bloodstream as CTCs, which are precursors of metastasis. Detecting these cells or their DNA (ctDNA) can therefore indicate metastatic potential [10]. MRD may also include micrometastases and small clusters of cancer cells in distant organs that are not yet detectable by imaging but that can later progress into overt metastases.

Cancers typically progress from benign to malignant. Benign tumors remain localized and cannot invade or spread to other parts of the body, whereas malignant tumors form metastases, which account for over 90% of cancer-related deaths [11]. The metastatic cascade comprises five steps: (1) cancer cell invasion and migration, (2) intravasation into the bloodstream or lymphatic system, (3) survival and transport through circulation, (4) extravasation into distant tissues, and (5) colonization and proliferation at secondary sites [12]. During this process, cancer cells disseminate to distant organs as CTCs [13]. In patients with metastatic cancer, CTCs are exceedingly rare, typically representing one cell per 10^9^ blood cells [14]. The acquisition of migratory and self-renewal (stem-like) properties by cancer cells is driven by epithelial–mesenchymal transition (EMT), a critical mechanism for metastasis. The expression of EMT markers, such as vimentin and N-cadherin, in CTCs is associated with increased metastatic potential and poor clinical outcomes [15,16]. The loss of epithelial characteristics and the acquisition of a mesenchymal-like transcriptional program are driven by the activation of the TGF-β, WNT, and NOTCH pathways, with regulation mediated by transcription factors such as ZEB, SNAIL, SLUG, and TWIST1 [17]. EMT-associated phenotypic plasticity may also complicate MRD detection, as tumor cells undergoing EMT frequently downregulate epithelial markers commonly used in CTC assays. Cells that successfully colonize distant organs are referred to as disseminated tumor cells (DTCs). DTCs can enter a prolonged non-dividing, dormant state that may persist for several years. DTCs in the BM serve as surrogates for MRD and are associated with the persistence of cancer cells after therapy, representing a strong indicator of poor prognosis. The potential activation of these dormant MRD cells is believed to trigger late-stage metastatic recurrence and tumor relapse. This metastatic process involves genetic and epigenetic alterations that occur throughout tumor initiation, promotion, and progression [18]. Genetic alterations play a pivotal role in enhancing the metastatic potential of tumors. For example, upregulation of mitogen-activated protein kinase kinase 21 has been shown to promote migratory and invasive phenotypes in breast cancer cells [19]. Epigenetic alterations involved in carcinogenesis include DNA methylation, histone acetylation, microRNAs, and nucleosome remodeling [20]. Abnormal DNA methylation—either hypomethylation or hypermethylation—is associated with mutations in tumor suppressor genes (TSGs) and proto-oncogenes. Hypermethylation of TSGs leads to transcriptional silencing, whereas hypomethylation activates proto-oncogenes and retrotransposons [21].

MRD represents a biologically intermediate state between primary tumor eradication and clinically detectable relapse [15]. During this stage, residual tumor cells frequently exist as DTCs in distant organs or as CTCs in peripheral blood. These cells may remain dormant for prolonged periods before reactivation and metastatic outgrowth. Importantly, both CTCs and DTCs represent cellular correlates of MRD and provide measurable biological substrates for molecular monitoring strategies.

The EMT plays a central role in this process. EMT-associated transcriptional programs, regulated by pathways such as TGF-β, WNT, and NOTCH, promote cellular plasticity, migratory capacity, immune evasion, and resistance to therapy [17]. Cells undergoing EMT often exhibit increased metastatic competence and are enriched among CTC populations detected in patients with cancer. These EMT-like cells may contribute to the persistence of MRD following systemic therapy.

From a clinical perspective, the biological processes underlying metastasis directly inform MRD detection strategies. For example, ctDNA reflects genomic alterations derived from both primary tumors and DTCs, while CTC-based assays can capture viable cells with EMT phenotypes that may not be detectable through conventional imaging. Therefore, MRD detection technologies serve as molecular proxies for the early stages of metastatic dissemination.

Understanding the biological relationship between metastasis and MRD is essential for the development of effective surveillance strategies. Early detection of MRD may enable therapeutic intervention before macroscopic metastases develop, providing a critical window for preventing relapse and improving long-term clinical outcomes.

## 3. MRD in Hematologic and Solid Tumors

MRD is a critical prognostic and predictive biomarker in hematologic malignancies such as acute lymphoblastic leukemia (ALL), chronic lymphocytic leukemia (CLL), and multiple myeloma (MM). In solid tumors, the application of MRD is more complex owing to spatial tumor heterogeneity and the absence of a uniform clonal marker.

### 3.1. In Hematologic Malignancies

Clinical studies have demonstrated that MRD negativity is strongly associated with improved progression-free survival (PFS) and overall survival (OS) [22]. The European Medicines Agency (EMA) recognizes MRD as a surrogate efficacy endpoint in clinical trials, which may accelerate drug approval [23]. Moreover, the European LeukemiaNet (ELN) provided updated guidelines (ELN-DAVID MRD Guideline, 2025 Update) to standardize thresholds and measurement timing based on specific molecular markers in patients with acute myeloid leukemia (AML), particularly when multiparameter flow cytometry (MFC) and quantitative real-time PCR (qPCR) are used for MRD detection [24].

#### 3.1.1. In AML

Numerous clinical trials have confirmed the prognostic importance of MRD negativity, which predicts significantly improved OS and lower relapse rates in patients treated with both intensive [9,25,26] and non-intensive chemotherapy regimens [27,28]. In AML, MRD is also used as a predictive marker to guide therapeutic decisions, specifically in determining whether allogeneic stem cell transplantation (alloSCT) offers greater benefits than consolidation therapy. Pasquini et al. [29] reported that MRD positivity before transplantation was significantly associated with higher relapse rates and poorer survival outcomes. This predictive role is further supported by studies suggesting that alloSCT should be mainly reserved for MRD-positive patients in first complete remission, even among favorable-risk AML subtypes [24].

Current limitations of molecular MRD assessment in AML are addressed in this study. Common biomarkers include t(15;17) promyelocytic leukemia (PML)-retinoic acid receptor alpha (RARA), t(8;21) AML1-RUNX family transcription factor 1 partner transcriptional co-repressor 1(RUNX1T1), B cell lymphoma2 (BCL2), fms-like tyrosine kinase 3 (FLT3), isocitrate dehydrogenase (IDH)1/2 and nucleophosmin (NPM1) [30]. qPCR is typically reliable only for monitoring the NPM1 gene and core-binding factor-mutated leukemia. To overcome challenges of standardization and inter-laboratory variability in other AML subtypes, Nachmias et al. [31] developed a new methodology: they created a synthetic minigene vector containing both Abelson tyrosine-protein kinase 1 (ABL1) and the sequence of the desired gene mutation, enabling objective and sensitive MRD evaluation. Inclusion of the ABL1 sequence permitted quantification of the mutation of interest using commercially available ABL1 standards for copy number estimation. This proof-of-concept test in patients with atypical RUNX1 and IDH1/2 mutations successfully demonstrated that MRD monitoring could detect relapse earlier than conventional flow cytometric assessments, particularly for atypical NPM1 and RUNX1 mutations.

Although its prognostic value is well established, the routine and standardized integration of MRD testing into surveillance and clinical management for every patient with AML is still evolving. MRD assessment is increasingly used for dynamic monitoring and treatment adjustment.

#### 3.1.2. In BCR/ABL1 and Philadelphia Chromosome-Positive Leukemia

Extensive data on MRD in BCR/ABL1 and Philadelphia (Ph) chromosome-positive leukemias primarily focus on chronic myeloid leukemia (CML) and Philadelphia chromosome-positive acute lymphoblastic leukemia (Ph-positive ALL). CML is defined by the presence of a Ph chromosome, resulting in a BCR/ABL1 gene fusion. MRD assessment in CML is standardized through measurement of BCR/ABL1 transcripts on the International Scale, which categorizes molecular responses and guides management, particularly regarding the potential for treatment-free remission.

Zuna et al. [32] performed a retrospective analysis of 147 children with BCR/ABL1-positive ALL treated between 2000 and 2021 to assess prognostic factors. A key finding was that MRD monitoring was a significant prognostic tool for typical ALL but not for patients with CML-like ALL (resembling CML crisis). They specifically compared outcome differences between the “CML-like” subtype and typical BCR/ABL1-positive ALL. Although OS was similar between the groups, patients with typical ALL experienced more relapses, whereas patients with CML-like ALL more frequently succumbed to the disease during the first remission. These findings indicate that MRD monitoring is crucial for guiding treatment intensity in ALL and highlight the need for distinct treatment approaches in future protocols for these biologically different subtypes.

The successful application of MRD monitoring in managing acute PML (APL) and CML has been reported by Chandhok et al. [33]. This success is largely due to both diseases being defined by specific, identifiable genetic translocations: the PML-RARα translocation in APL and the BCR-ABL translocation in CML. MRD assessment, primarily through highly reliable qPCR testing, has become integral to clinical practice for both conditions, guiding crucial decisions, such as treatment duration, evaluation of treatment effectiveness, and prediction of potential relapse. Furthermore, MRD monitoring has reduced the need for frequent invasive procedures in APL and informs decisions regarding therapy modification or eligibility for treatment-free remission in CML. Consequently, MRD assessment is now firmly incorporated into major international treatment guidelines for leukemia.

#### 3.1.3. In MM

MRD plays a critical role in the management and treatment of MM. Chen et al. [34] noted that despite improvements in MM therapies, complete eradication of malignant plasma cells remains difficult, making MRD monitoring essential for evaluating treatment success and predicting patient prognosis. They highlighted the superior sensitivity and applicability of newer methods, such as next-generation sequencing (NGS). A large meta-analysis of >8000 patients with MM confirmed the positive impact of MRD negativity on long-term survival outcomes [35]. Moreover, Anderson et al. [36] discussed the evolving role of MRD in MM treatment and new drug registration. They highlighted emerging blood-based assays, including mass spectrometry and cell-free DNA (cfDNA), as less invasive complementary tools and demonstrated the clinical utility of MRD across various patient scenarios throughout the disease spectrum. The treatment landscape for MM has been fundamentally transformed by the development of novel therapeutic agents, making the achievement of an MRD-negative response a viable goal for patients with newly diagnosed and relapsed/refractory diseases. Current standard practice for MRD assessment relies on NGS and next-generation flow for BM analysis, supplemented by positron emission tomography/computed tomography for extramedullary disease. However, the field is rapidly evolving, with emerging technologies such as mass spectrometry, cfDNA analysis, and advanced imaging poised to redefine future MRD assessment strategies.

Across acute leukemia, chronic leukemia, lymphoma, and myeloma, MRD positivity after treatment is consistently associated with a higher relapse risk and inferior survival, whereas MRD negativity predicted better outcomes. For example, in AML, 5-year OS can be approximately halved in patients who remain MRD-positive compared with those who become MRD-negative after achieving remission [8,33,37,38]. The detection and monitoring of MRD are therefore indispensable in managing hematological malignancies, as they provide high-fidelity information that accurately predicts long-term patient outcomes and informs subsequent therapeutic strategies.

Altogether, measurable MRD in hematological malignancies functions as an established fundamental prognostic marker, with critical predictive value for guiding individualized therapeutic decisions across various disease types. MRD negativity is consistently and strongly associated with improved PFS and OS, whereas MRD positivity after treatment is uniformly linked to a higher relapse risk and inferior survival outcomes. The clinical utility of MRD is recognized by major regulatory bodies, such as the EMA, which supports MRD as a surrogate efficacy endpoint in clinical studies.

#### 3.1.4. MRD in Lymphoma

MRD monitoring has also become an active area of research in lymphoid malignancies, particularly diffuse large B-cell lymphoma (DLBCL) and follicular lymphoma [7]. Advances in high-throughput sequencing technologies have enabled the identification of patient-specific immunoglobulin gene rearrangements and tumor-derived mutations that can be tracked in plasma using ctDNA-based assays [7].

In DLBCL, a study has demonstrated that molecular MRD detection using NGS of immunoglobulin gene rearrangements or tumor-specific mutations can predict relapse earlier than conventional imaging modalities [7]. Persistent or re-emerging ctDNA after treatment has been associated with inferior PFS and may identify patients who could benefit from early therapeutic intervention.

Similarly, in follicular lymphoma, molecular monitoring of B-cell receptor clonotypes has shown prognostic value for predicting relapse following immunochemotherapy or stem cell transplantation. MRD assessment using sensitive sequencing approaches can detect residual disease at levels far below the threshold of radiologic detection.

These findings highlight the growing clinical importance of MRD monitoring in lymphoma and suggest that integration of molecular MRD testing into clinical trials may facilitate response-adapted treatment strategies in the future.

### 3.2. In Solid Tumors

Advances in liquid biopsy technologies have enabled the detection of ctDNA for MRD assessment in cancers such as colorectal cancer (CRC), non-small cell lung cancer (NSCLC), and breast cancer [39,40]. ctDNA-based MRD monitoring provides dynamic, non-invasive surveillance and can predict disease recurrence months before it becomes detectable by imaging [41].

#### 3.2.1. CRC

Liquid biopsy approaches using stool and plasma samples have emerged as promising tools for cancer detection. In particular, stool-based assays are effective for CRC detection because genomic and epigenomic alterations arising during CRC tumorigenesis can be readily identified in stool samples. Methylation signatures in tumor-derived DNA obtained from stool or blood enable early cancer detection [42]. Stool-based biomarkers, including multigene fecal DNA methylation panels and microbiome-associated markers, have demonstrated high sensitivity in identifying residual or recurrent diseases, especially in early-stage or localized tumors. A panel of three methylated gene markers—syndecan2 (SDC2), alcohol dehydrogenase iron containing 1 (ADHFE1), and protein phosphatase 2 regulatory subunit b gamma (PPP2R5C)—showed a sensitivity of 87.1% for CRC detection, with higher sensitivity for distal CRC than for proximal CRC [43,44].

For instance, ctDNA-positive patients showed a 43.5-fold higher risk of CRC recurrence than ctDNA-negative patients [45]. Multiple studies have shown that postoperative ctDNA positivity after curative surgery identifies a subgroup with a very high recurrence risk and can stratify benefit from adjuvant chemotherapy, leading to ongoing trials of ctDNA-guided treatment escalation or de-escalation. Similar retrospective and prospective data in lung, breast, bladder, and other cancers indicate that ctDNA-defined MRD positivity after definitive local therapy is associated with markedly worse recurrence-free survival and OS and often precedes radiologic relapse by several months [31,46].

Notably, ctDNA levels can be transiently elevated immediately post-surgery owing to the release of tumor DNA during surgical manipulation; therefore, most studies recommend delaying MRD assessment beyond the first few postoperative days to avoid false-positive results. When MRD is present, ctDNA-based assays typically detect recurrence a median of several months earlier (often 6–8 months in some series) than conventional imaging or clinical symptoms in localized disease, offering a potential window for earlier intervention [46,47].

In contrast, a real-world study reporting the utility and performance of ctDNA monitoring, specifically the K-track assay, was performed by Hoang et al. [48] to predict cancer recurrence and treatment response, primarily in a Southeast Asian patient population. The researchers analyzed 623 patients with various solid tumors (stages I–IV) in Vietnam to assess how ctDNA testing is utilized in clinical practice and to determine its prognostic value for predicting recurrence, often months before detection by imaging. The key findings indicated that postoperative ctDNA positivity significantly increased the risk of recurrence across multiple cancer types, demonstrating the high sensitivity and specificity of the assay. The authors concluded that ctDNA serves as a strong prognostic biomarker for cancer management, although its real-world implementation is often limited by factors such as high costs and logistical challenges related to sample quality.

In patients with CRC who have undergone definitive therapy, recurrence of advanced-stage disease is common, often occurring within 2 years of surgery [49]. Several studies have shown that ctDNA-based MRD detection is highly sensitive and specific for predicting relapse, providing a lead time significantly longer than that of conventional markers, such as carcinoembryonic antigen [39,50,51]. These studies employed specific commercial ctDNA assays, including Signatera™ and Guardant Reveal™, to assess recurrence risk and showed that integrating serial testing with epigenomic signatures can further enhance ctDNA sensitivity. In stage I–III CRC, longitudinal tumor-informed ctDNA assays have reported median lead times of roughly 8–9 months between the first ctDNA detection and radiographic recurrence, with individual ranges extending from <1 month to approximately 16–17 months [39,52]. Ultimately, ctDNA status serves as a powerful independent predictor of recurrence-free survival in both non-metastatic CRC and resectable colorectal liver metastasis.

#### 3.2.2. NSCLC

Abbosh et al. [53] reported a phylogenetic approach for analyzing ctDNA to monitor NSCLC progression and recurrence. This study was part of the larger TRACERx (Tracking NSCLC Evolution Through Therapy) study, in which patient-specific ctDNA was profiled using multiplex-PCR NGS to track tumor evolution following primary surgery. The findings identified several clinicopathological predictors of ctDNA detection, including non-adenocarcinoma histology and tumor necrosis, and established a correlation between tumor volume and ctDNA variant allele frequency. Crucially, longitudinal analysis demonstrated that this phylogenetic ctDNA profiling could predict and characterize NSCLC relapse, often providing a significant lead time before clinical confirmation, and could even track resistance to adjuvant chemotherapy. Overall, the findings support the use of ctDNA platforms to guide therapeutic studies by identifying residual disease and targeting emerging subclones in the adjuvant setting.

A meta-analysis by Lu et al. [54] evaluated the effectiveness of ctDNA detection for predicting recurrence in patients with early-stage NSCLC after surgery. They systematically reviewed 30 publications to assess the utility of ctDNA-based MRD testing for both diagnosis and survival prognosis. A core finding is that longitudinal ctDNA monitoring substantially improves predictive sensitivity compared with single fixed-time point (landmark) testing. The analysis also compared two main strategies, tumor-informed and tumor-agnostic, and found that, although the tumor-informed approach excels in specificity, the tumor-agnostic method often provides higher sensitivity and identifies more clinically actionable genetic variants over time. The authors concluded that ctDNA-MRD testing offers clinically significant prognostic information and should be tailored to individual patient needs.

In resected NSCLC, early studies reported median ctDNA lead times of approximately 2–5 months before radiologic relapse, with ranges from approximately 10 days to nearly 1 year, depending on the assay and follow-up. Updated analyses using more sensitive technologies have reported median lead times of approximately 5 months and sensitivities around 80%, confirming that MRD-positive lung cancer almost invariably relapses despite apparently clean imaging.

#### 3.2.3. Breast Cancer

In early-stage breast cancer, serial personalized ctDNA monitoring detected metastatic recurrence with a median lead time of approximately 8–9 months, with some patients showing ctDNA positivity for more than 1–2 years before overt relapse. These studies show that ctDNA-based MRD can be intermittently detectable, and lead time varies by subtype—often longer in estrogen receptor (ER)-positive disease—but persistent or rising ctDNA strongly correlates with eventual relapse [55,56].

Eliott et al. [56] conducted a longitudinal evaluation of ctDNA as an emerging biomarker in patients with early breast cancer receiving neoadjuvant therapy. The authors employed a highly sensitive tumor-informed assay to assess its clinical validity and prognostic value in a real-world cohort. The findings indicate that ctDNA detection at baseline is frequent and associated with a shorter recurrence-free interval, with persistent ctDNA detection midway through neoadjuvant therapy serving as a strong predictor of disease recurrence, especially in human epidermal growth factor receptor (HER)2-negative cases. HER2, a member of the epidermal growth factor receptor (EGFR) family, regulates cell growth, cell cycle progression, and survival, and its overexpression increases tumor aggressiveness, making its detection vital for treatment decisions [57]. Notably, ctDNA detection post-surgery or during follow-up demonstrated a 100% positive predictive value for subsequent recurrence, highlighting its potential utility for risk monitoring. This study supports prospective trials integrating sensitive ctDNA testing to guide individualized treatment strategies [56].

In other tumor types, such as bladder, gastric, liver, and ovarian cancers, emerging data suggest similar or slightly shorter median lead times, generally ranging from 3 to 6 months, with substantial inter-patient variability. Factors influencing lead time include tumor biology and DNA shedding (e.g., low-shedding or sanctuary sites can shorten or eliminate lead time), assay sensitivity (tumor-informed vs. agnostic), and sampling frequency, as infrequent sampling can underestimate the true interval between molecular and clinical relapses [48,55]. Despite these promising results, challenges remain in standardizing assay sensitivity, interpretation, and timing for patient-informed MRD assessments in solid tumors.

## 4. Techniques for MRD Detection

Advances in highly sensitive molecular technologies have significantly improved the ability to detect MRD. In particular, ctDNA analysis through liquid biopsy enables non-invasive monitoring of tumor-specific mutations in the bloodstream [39,53]. These approaches allow clinicians to detect molecular relapse months before radiologic evidence of disease recurrence becomes apparent [50].

Several advanced technologies have been developed for MRD detection, including MFC, quantitative PCR, NGS, and digital PCR [9,35]. These approaches differ in sensitivity, specificity, and clinical application, and their implementation depends on disease type, available biomarkers, and logistical feasibility. As technology continues to evolve, multi-platform approaches may offer the most comprehensive MRD assessment [9,35].

### 4.1. Conventional Techniques

In addition to qPCR, which remains a reliable tool for monitoring MRD and assessing treatment responses, advanced technologies with high sensitivity have further enhanced MRD detection and analysis. One such technology is NGS, a powerful tool applicable across both hematologic malignancies and solid tumors. NGS enables comprehensive characterization of clonal mutations, chromosomal alterations, and gene fusions at deep sequencing depths, allowing the detection of rare residual tumor cells at levels as low as 10^−5^–10^−6^ [58]. Furthermore, NGS-based assays are patient-specific while capturing the genetic heterogeneity of cancer, which is critical for monitoring therapy resistance. Notably, NGS is used for relapse prediction and to guide personalized treatment decisions, including identifying actionable mutations for targeted therapies [59].

Additionally, dPCR is a high-performance technology that allows absolute quantification of nucleic acids. Two main types of dPCR are available: (i) chip-based dPCR, which uses microfluidic devices to partition the lipid bilayer chamber system and nanoliter self-priming compartmentalization chip, and (ii) droplet-based dPCR (ddPCR), which partitions a sample into thousands of individual droplets. ddPCR has been widely used for ctDNA detection and monitoring in patients with cancer, detecting 62.5% of tumor protein p53 (TP53) mutations in patients with ovarian cancer, 83.1% of phosphatidylinositol 3-kinase catalytic subunit alpha (PIK3CA) mutations in patients with breast cancer, and 86.3% of EGFR mutations in patients with NSCLC [60,61,62]

Single-cell RNA sequencing (scRNA-seq) has emerged as a transformative tool for understanding MRD complexity, enabling profiling of individual tumor cells and revealing the diverse genetic and functional landscapes hidden within bulk samples. For example, scRNA-seq provides a comprehensive map of tumor heterogeneity in MM by analyzing thousands of cells independently, thereby detecting rare malignant cell populations often missed by bulk RNA sequencing methods [63]. Matera et al. [64] demonstrated the feasibility of using scRNA-seq to identify patient-specific clonotypic V(D)J rearrangements, which serve as molecular fingerprints to track residual tumor cells post-treatment in MM. By enriching scRNA-seq libraries with V(D)J sequences, the authors reliably identified residual malignant plasma cells and analyzed their gene expression profiles and surface protein markers in detail. Combining conventional scRNA-seq with V(D)J library enrichment presents a practical solution, as it does not require additional tissue input, remains cost-effective, and simplifies data analysis workflows, making it highly suitable for routine MRD investigations in MM.

Another technology used to detect and quantify MRD is MFC. MFC enables the identification of abnormal cell populations based on immunophenotypic markers in various hematological malignancies. In hematologic cancers such as ALL or CLL, MFC can detect MRD with high sensitivity (10^−5^ to 10^−6^) by analyzing surface and intracellular antigens [65]. Recent advances in standardized antibody panels and automated analyses have improved the reproducibility and clinical utility of this technique [66]. Fürstenau et al. [67] evaluated MRD in CLL following highly effective combination treatments using ctDNA analysis compared with multicolor flow cytometry, which is less effective at detecting disease in the lymph nodes. The authors tracked patient-specific V(D)J rearrangements and somatic driver mutations in blood plasma using ddPCR during the CLL2-BAAG trial (NCT03787264) and systematically compared the results with matched flow cytometry data. Although the two methods showed high overall concordance, ctDNA detected residual disease in a significant portion of samples deemed undetectable by flow cytometry, particularly in patients with predominantly nodal residual disease. Therefore, the authors concluded that ctDNA-based MRD assessment is a promising complement to cell-based methods, such as flow cytometry, for reflecting the total disease burden across different compartments.

### 4.2. Emerging Methods and Multi-Omics Integration

Recent advances in precision medicine underscore the importance of integrating comprehensive molecular data to better understand cancer biology and improve clinical outcomes. Lim et al. [68] reviewed the latest advancements in single-cell omics and multi-omics technologies for high-resolution molecular profiling. Single-cell omics allows the analysis of individual cells to uncover cellular diversity and dynamic states across various layers, including the genome, transcriptome, proteome, methylome, chromatin accessibility, chromatin conformation, and histone modifications. Single-cell multimodal omics has further evolved by combining multiple omics layers to provide a holistic view of complex cellular processes [68]. This systems-level approach facilitates the identification of novel biomarkers, elucidation of dysregulated pathways, and prediction of therapeutic responses across cancer types [69]. Integrating multi-omics with liquid biopsy has enhanced the sensitivity and specificity of cancer diagnostics and monitoring [70]. By combining multi-layered molecular profiles with non-invasive sampling, this approach enables personalized assessment of tumor evolution. Together, multi-omics and liquid biopsy represent a transformative strategy in oncology, advancing patient-informed MRD for individualized cancer care.

Integrative multi-omics approaches have played key roles in refining AML risk stratification and MRD monitoring by combining RNA, proteomic, and ctDNA data. New methods leveraging multi-omics have been developed to improve MRD detection in patients with AML. Saeed et al. [71] examined the effectiveness of ultra-deep sequencing (UDS) as a highly sensitive method for detecting MRD in pediatric AML and compared it with conventional MFC. The authors first used WES to identify leukemia-specific mutations in diagnostic samples and then applied UDS to track these mutations during treatment, finding that UDS was significantly more sensitive than MFC. Over half of the samples negative by MFC were positive using UDS, demonstrating the ability of this molecular method to detect lower levels of MRD. Furthermore, single-cell multi-omics analysis characterized the genomic and protein signatures of the residual mutated cells, confirming the UDS findings and showing that these cells could be either founding stem/progenitor cells or mature leukemic cells. These results support the use of UDS as a sensitive tool for MRD detection in pediatric AML.

However, Thompson et al. [72] introduced a novel multi-omics single-cell MRD (scMRD) assay designed to overcome the limitations of existing methods for detecting MRD in AML. This scMRD assay, with a 0.01% limit of detection, utilizes a multistep pipeline that includes blast cell enrichment and a specialized DNA and protein panel to provide comprehensive single-cell clonal architecture and immunophenotyping. Using Mission Bio technology, the assay identifies and correlates co-occurring variants, reducing false positives compared to bulk assays, and can construct phylogenetic trees of detected cells. The integration of genotypes and immunophenotypes further enhances MRD detection by revealing genotype-specific protein expression patterns. Its robust performance and reproducibility were confirmed in both surrogate samples and samples from patients with AML, suggesting its potential as a scalable tool for guiding patient stratification and therapeutic decisions.

In a review article, Sanches et al. [73] focused on strategies for integrating multi-omics data, specifically transcriptomics, proteomics, and metabolomics, to achieve a comprehensive understanding of biological systems. The authors detailed various computational techniques, such as co-expression analysis, network construction (e.g., gene–metabolite networks), and genome-scale metabolic models, which facilitate the identification of complex biological patterns and interactions often missed by single-omics analyses. A novel approach for cancer monitoring was described by Widman et al. [74], who introduced MRD-Enrichment and detection using Genomic Enrichment (EDGE), a platform that utilizes machine learning and whole-genome sequencing (WGS) to enhance ctDNA detection for assessing MRD and therapeutic response. The authors evaluated MRD-EDGE^SNV^ for single-nucleotide variant detection using deep learning and MRD-EDGE^CNV^ for copy number variant detection, which integrates read depth, B-allele frequency, and fragment length entropy. The MRD-EDGE approach offers several clinically significant applications and demonstrates comparative performance benefits, particularly in low-tumor-fraction settings. They demonstrated the high sensitivity of the platform for detecting ctDNA across various clinical contexts, including early-stage and metastatic cancers, as well as following surgery in multiple cancer types, such as CRC, triple-negative breast cancer, and NSCLC. For therapeutic response monitoring, MRD-EDGE could track changes in the tumor fraction in response to neoadjuvant immunotherapy in NSCLC, as well as in advanced melanoma and small cell lung cancer. Notably, its high sensitivity enabled early lesion detection by identifying ctDNA shedding from precancerous colorectal adenomas and pT1 carcinomas (polyp cancers), suggesting that dysplastic tissue may release ctDNA even in the absence of a significant invasive component. This finding supports the feasibility of ctDNA-guided detection of premalignant lesions. Furthermore, the authors showed that combining WGS breadth with machine learning-guided signal enrichment improved MRD-EDGE performance, overcoming sensitivity limitations associated with deep targeted sequencing panels, and enhancing prior WGS-based approaches.

Artificial intelligence (AI) techniques are increasingly applied to the study of MRD, particularly in hematological malignancies such as MM and AML. These approaches enhance the reliability of MRD detection and facilitate prognostic prediction. Integrating genomic, transcriptomic, and proteomic data with AI enables a more comprehensive understanding of MRD biology and supports the identification of novel biomarkers and therapeutic targets. Machine Learning and deep learning methods have been used to analyze high-dimensional data generated by flow cytometry, NGS, and PCR, thereby improving the sensitivity and specificity of MRD detection. Moreover, mixture models and feature-agnostic transformer-based encoders have been applied to flow cytometry data to automate MRD assessment while providing interpretable and clinically actionable results [75].

Notably, AI models integrate clinical, genetic, and immune-related biomarkers to predict which patients are likely to achieve MRD negativity, a key prognostic factor for risk stratification and personalized treatment planning. Martinez-Lopez et al. utilized NGS combined with AI to categorize patterns of MRD evolution over time in 482 patients with MM. They demonstrated that greater clonal diversity of immunoglobulin genes, which may reflect immune recovery, is associated with a favorable prognosis in MM. These results suggest that integrating MRD dynamics with clonal diversity assessments can accurately predict disease progression and inform clinical decisions [76].

Mocking et al. developed a fully automated, interpretable, and objective computational pipeline to assess MRD in AML using Gaussian mixture models (GMMs). This method reduced analysis time from 30 min to 3 s while maintaining high accuracy, demonstrating that GMM-based computational pipelines can provide a robust, rapid, and interpretable alternative to manual MRD assessment in AML, with the potential for broader clinical adoption [75].

In addition, AI can be applied for dynamic MRD monitoring by analyzing longitudinal MRD data to predict relapse risk and optimize therapeutic strategies, such as adjusting consolidation therapy in leukemia or MM [75,76].

**Figure 1:**
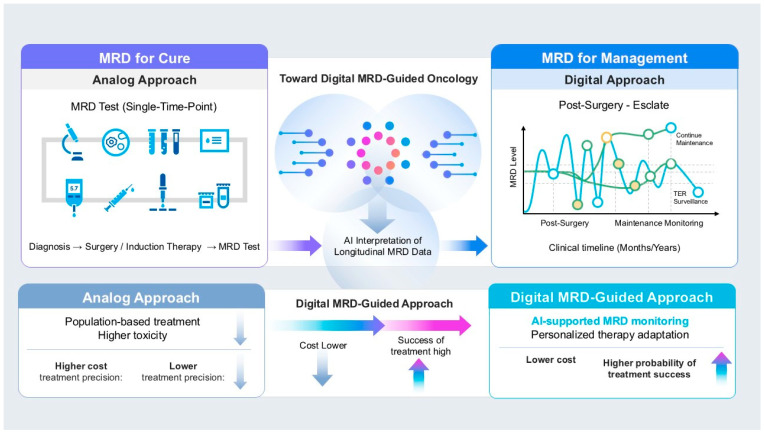
Evolution of MRD-guided cancer management from analog treatment strategies to digital longitudinal monitoring. The figure illustrates the conceptual shift in oncology from conventional minimal residual disease (MRD) assessment to longitudinal MRD-guided disease management. In the traditional analog approach (left panel), MRD is typically measured at a single clinical time point, such as after surgery or induction therapy, providing limited information on residual disease dynamics. Advances in highly sensitive molecular technologies, including circulating tumor DNA (ctDNA) analysis, now enable longitudinal monitoring of MRD over time (middle panel). Integration of these data with computational and artificial intelligence (AI)-based analysis supports dynamic interpretation of MRD trajectories. In the digital MRD-guided approach (right panel), MRD levels are monitored along the clinical timeline (months to years), enabling early detection of molecular relapse and adaptive clinical decision-making, such as therapy escalation, continuation of maintenance therapy, or surveillance during treatment-free remission. Collectively, these innovations highlight a pivotal shift toward highly sensitive, high-resolution molecular diagnostics that support individualized patient management. As multi-omics technologies continue to mature, their integration into routine clinical workflows promises to refine prognostication, optimize therapeutic interventions, and advance the delivery of personalized cancer care (Table 1).

Figure 1 illustrates a paradigm shift in cancer therapy in which the role of MRD has evolved from a single-time-point assessment aimed at curing (“MRD for Cure”) to a framework for longitudinal MRD-guided disease management. Conventional analog treatment strategies rely on uniform drug administration, often resulting in increased costs and treatment-related toxicity. In contrast, serial MRD measurements generate digital molecular data that enable continuous monitoring of disease status over time. The integration of these data with AI-driven models allows accurate risk stratification and personalized clinical decision-making, including treatment escalation, de-escalation, or surveillance. This digital MRD-guided approach improves treatment efficacy while reducing unnecessary interventions and costs, highlighting MRD as a central driver in the transition from cure-oriented cancer therapy to precision management-focused oncology.

**Table 1:** Characteristics of techniques used for MRD detection.

MRD Method	Sample Type	Typical Sensitivity	Primary Clinical Use	Strengths	Limitations	Current Clinical Actionability	References
Multiparameter Flow Cytometry (MFC)	Bone marrow, blood	10^−4^–10^−5^	Hematologic malignancies (ALL, AML, CLL, MM)	Broad applicability; rapid turnaround	Lower sensitivity than molecular methods; inter-lab variability	High in hematologic malignancies (risk stratification, transplant decisions)	[65]
RT-qPCR	Bone marrow, blood	10^−5^–10^−6^	Fusion genes/recurrent mutations (e.g., BCR-ABL1, NPM1)	High sensitivity; standardized for selected targets	Limited to known targets; relative quantification	High for selected molecular subtypes	[77]
Next-Generation Sequencing (NGS)	Bone marrow, blood, plasma	10^−5^–10^−6^	Hematologic and solid tumors	Detects clonal heterogeneity; tumor-informed assays	Cost; bioinformatics complexity; clonal hematopoiesis confounding	High (hematologic); Moderate (solid tumors)	[58]
Digital PCR (ddPCR/cdPCR)	Blood, plasma	10^−4^–10^−5^	Targeted mutation tracking	Absolute quantification; high precision	Limited multiplexing; target-restricted	Moderate–High for known mutations	[60,61,62]
ctDNA (tumor-informed)	Plasma	10^−5^–10^−6^	Solid tumor MRD detection	High specificity; early relapse detection	Requires tumor tissue; cost	Moderate (mainly within trials)	[78]
ctDNA (tumor-agnostic)	Plasma	10^−3^–10^−5^	Broad surveillance, variant discovery	No tumor tissue required; broader mutation capture	Lower specificity for MRD	Low–Moderate	[78]
Single-cell sequencing (scRNA-seq/scMRD)	Bone marrow, tissue	<10^−6^ (theoretical)	Clonal architecture, resistant subpopulations	Ultra-high resolution; biological insight	Cost; scalability; limited clinical validation	Exploratory	[64]
Multi-omics/AI-integrated platforms	Plasma, tissue	Variable	Advanced MRD detection and prediction	Signal enhancement; low tumor fraction detection	Regulatory and interpretability challenges	Exploratory/Early clinical	[75,76]

Abbreviations: MRD, Minimal Residual Disease; ALL, Acute Lymphoblastic Leukemia; AML, Acute Myeloid Leukemia; CLL, Chronic Lymphocytic Leukemia; MM, Multiple Myeloma; RT-qPCR, Reverse Transcription Quantitative Polymerase Chain Reaction; ddPCR, Droplet-Based Digital PCR; cdPCR, Chip-based Digital PCR; ctDNA, Circulating Tumor DNA; scRNA-seq, Single-cell RNA Sequencing; scMRD, Single-cell MRD assay; AI, Artificial Intelligence.

## 5. Eradicating MRD Positivity

Treatments aimed at eradicating MRD positivity are mainly described in hematologic malignancies and fall into several major categories: MRD-directed immunotherapies (particularly in B-ALL), hypomethylating agents and other “pre-emptive” therapies in AML/myelodysplastic syndromes, post-transplant immunomodulation (e.g., donor lymphocyte infusion, interferon), and MRD-adapted intensification or switching of systemic therapy. In solid tumors, MRD is typically detected as ctDNA after surgery, and current strategies focus on escalation or prolongation of adjuvant systemic therapy within clinical trials rather than the use of specific MRD-targeted drugs.

### 5.1. Immunotherapy

MRD represents a critical juncture in cancer treatment, marking the transition from measurable disease to potential cure. Integrating immunotherapy into MRD-driven treatment strategies offers a promising approach for eradicating residual malignant cells and achieving durable remission. MRD positivity after initial therapy serves as a biomarker to escalate or introduce immune-based treatments. For example, in head and neck cancer, MRD detection via ctDNA allows immune checkpoint blockade to predict treatment response and recurrence risk [79]. The curative potential of immunotherapy is enhanced when guided by patient-informed MRD, enabling timely and personalized interventions aimed at complete disease eradication. Moreover, MRD negativity after immunotherapy is increasingly regarded as a surrogate endpoint for cure, particularly in relapsed/refractory MM [80]. Future therapeutic paradigms may rely on patient-informed MRD monitoring to optimize immunotherapy regimens and minimize overtreatment. Notably, no cancer currently has a drug with a regulatory label explicitly requiring treatment initiation, discontinuation, escalation, or de-escalation based on MRD results, even though MRD is widely used in practice and clinical trials across many malignancies.

MRD plays an increasingly complex role in hematological malignancies, specifically ALL, AML, and MM. Initially, MRD clearance—for example, with the Food and Drug Administration (FDA)-approved agent blinatumomab, an anti-CD19 Bispecific T-cell engager in ALL—was strongly correlated with improved outcomes [81,82,83]. However, recent findings complicate the relationship between MRD status and survival, suggesting that drug mechanisms may extend beyond simple MRD clearance, or that the biological heterogeneity of diseases such as ALL challenges straightforward biomarker interpretation [84].

In contrast, inotuzumab ozogamicin (INO) and CD19 chimeric antigen receptor (CAR) T cells are used to eradicate MRD in B-cell ALL (B-ALL), demonstrating high MRD clearance rates and increasingly being administered earlier in the disease course to eliminate residual disease. Short et al. [85] reported a retrospective analysis of a dose-dense treatment regimen involving 21 patients with B-ALL, combining mini-hyperfractionated cyclophosphamide, vincristine, and dexamethasone, alternating with mini-methotrexate and cytarabine chemotherapy, INO, and blinatumomab, administered earlier than standard practice. The authors found that this therapy was safe and achieved rapid, deep MRD negativity in all responding patients, often after only one cycle. They concluded that this regimen warrants further prospective evaluation owing to the high rates of early MRD clearance and favorable 1-year OS observed in both frontline and MRD-positive patient groups [85].

Moreover, a clinical trial investigating a novel CAR T-cell therapy for relapsed or refractory B-ALL was conducted by Ghorashian et al. [86]. This study included 12 heavily pretreated pediatric patients and found that the dual-targeting approach was well tolerated, with no severe cytokine release syndrome, and successfully induced MRD-negative complete remission in 83% of patients. Crucially, with a median follow-up of 8.7 months, no cases of relapse owing to antigen-negative escape were recorded, suggesting that dual targeting may effectively prevent this form of treatment failure, although longer CAR-T cell persistence remains a goal for future optimization.

In contrast, the effectiveness of various Bispecific Antibody (BiAb) therapies in achieving MRD negativity is generally high, with several products demonstrating particularly strong MDS clearance in patients who achieve a complete or better response [87]. Data from the MajesTEC-1 (NCT03145181/NCT04557098), MonumenTAL-1 (NCT03399799/NCT04634552), MagnetisMM-1/3 (NCT04649359), and LINKER-MM1 (NCT03761108) clinical trials indicate robust MRD negativity rates for BiAbs such as B-cell maturation antigen (BCMA)-targeted Teclistamab, Elranatamab, Linvoseltamab, and G protein-coupled receptor, class C, group 5, member D (GPRC5D)-targeted Talquetamab [88,89,90]. BCMA and GPRC5D are cell surface transmembrane proteins expressed on multiple cell types and serve as immunotherapeutic targets for MM. CD3, a protein complex on the surface of T cells, mediates the cytotoxic killing of cancer cells by simultaneously binding BCMA or GPRC5D on myeloma cells and CD3 on T cells. This interaction triggers T-cell activation, proliferation, and the release of cytotoxic molecules (such as perforin and granzyme), directly killing myeloma cells [88,89]. This approach can induce deep and sustained responses, including the eradication of MRD [87].

### 5.2. Targeted Therapies

Oral azacitidine (oral-AZA), also known as CC-486, has demonstrated positive effects on MRD outcomes in patients with AML in first remission after intensive chemotherapy. Döhner et al. [91] reported that oral-AZA therapy conferred OS and/or recurrence-free survival (RFS) benefits, even in patients with adverse prognostic features and MRD positivity. Maintenance therapy with oral-AZA was associated with a higher rate of MRD response—defined as conversion from MRD-positive at baseline to MRD-negative—in patients harboring NPM1 or FLT3 mutations compared with placebo.

Overall, these findings suggest that oral-AZA prolongs OS and RFS independent of the initial MRD status and actively promotes MRD clearance and sustains MRD negativity in patients with AML.

Levis et al. [92] reported results for quizartinib, a highly selective type II FLT3 inhibitor, in the phase 3 trial QuANTUM-First trial (NCT02668653), which investigated the upfront addition of quizartinib to intensive chemotherapy in patients with FLT3-internal tandem duplication (ITD) mutant AML [92]. The addition of quizartinib improved OS compared with placebo, and patients receiving quizartinib had lower post-induction FLT3-ITD NGS-MRD levels than the placebo group. However, although a higher proportion of patients achieved undetectable MRD with quizartinib, this deeper clearance did not appear to confer a significant survival benefit. Furthermore, quizartinib, along with other FLT3 inhibitors such as gilteritinib and sorafenib, have been used as MRD-guided salvage therapy for molecular failure in patients with AML and baseline FLT3-ITD mutations. In one cohort of 48 patients treated with these FLT3 inhibitors for MRD failure, an overall MRD response was achieved in 64%, with 40% of evaluable patients reaching MRD negativity, resulting in an estimated 2-year OS of 80% [93,94].

A phase 2 clinical study (Clinicaltrials.gov #NCT04113018) published by Bhutani et al. [95] evaluated an MRD-driven treatment strategy for newly diagnosed MM using the combination of daratumumab, carfilzomib, lenalidomide, and dexamethasone (Dara-KRd) as induction therapy, followed by treatment escalation or de-escalation based on each patient’s MRD status. This regimen achieved high rates of stringent complete responses and MRD negativity, which improved over time, supporting the use of MRD-adapted post-induction strategies. Correlative analyses of immune cell activity demonstrated that the Dara-KRd combination strongly activated memory T cells, which are associated with MRD negativity. The authors concluded that these findings support the need for randomized trials comparing upfront autologous stem cell transplantation (ASCT) versus no ASCT for MRD-negative patients and for treatment escalation for MRD-positive patients.

Therapeutic approaches are often combined or tailored based on cancer type, patient risk factors, and response to initial treatment. The overarching goal is to achieve and sustain MRD negativity, which is strongly associated with improved survival and reduced relapse rates.

## 6. Limitations

MRD detection has significantly transformed the management of hematological malignancies by providing a sensitive tool for risk stratification and treatment monitoring. However, its predictive value remains imperfect. MRD positivity after therapy does not always lead to clinical relapse, and MRD negativity does not guarantee complete disease eradication. Chandhok et al. and Chen et al. [33,96] evaluated the current utility and limitations of MRD testing in the diagnosis and treatment of both hematological and solid cancers. They highlighted that, although MRD testing is useful for risk stratification, especially in some acute leukemias, its predictive value is often variable and insufficiently accurate to reliably identify patients who will relapse. MRD positivity after therapy does not always predict relapse, and MRD negativity does not guarantee a cure. Despite the enthusiasm surrounding MRD, its complex use and inherent limitations can result in high false-positive and false-negative rates, calling into question the robustness of many MRD-guided therapeutic interventions. Additionally, the presence of low-level residual blasts may not always reflect true MRD, leading to potential misinterpretation. This creates limitations in clinical management and in the use of MRD as a therapeutic target within personalized medicine, with these challenges being closely interdependent.

Pre-analytical standardization represents a critical requirement for reliable MRD assessment. Variability in sample collection, processing time, storage conditions, and nucleic acid extraction methods can significantly affect the sensitivity and reproducibility of MRD assays [97,98]. For ctDNA-based MRD detection, factors such as plasma separation timing, stabilization of blood samples, and avoidance of leukocyte lysis are particularly important because contamination with genomic DNA may dilute tumor-derived signals [99]. International initiatives are therefore working toward standardized protocols for sample handling, analytical validation, and reporting frameworks to ensure inter-laboratory comparability of MRD results [100].

Another important biological confounder in MRD testing, particularly for solid tumors, is clonal hematopoiesis of indeterminate potential (CHIP). Age-related somatic mutations occurring in hematopoietic stem cells, commonly affecting genes such as *DNMT3A*, *TET2*, and *ASXL1*, can release mutant DNA fragments into the circulation that may be incorrectly interpreted as tumor-derived ctDNA [101,102]. Without appropriate filtering strategies, CHIP-associated variants may lead to false-positive MRD detection. To address this challenge, several assay designs incorporate matched white blood cell sequencing or bioinformatic filtering approaches to distinguish tumor-derived mutations from hematopoietic clones [103]. Careful consideration of CHIP is therefore essential when interpreting MRD results, particularly in plasma-based assays for solid tumors.

Current methodologies for MRD detection primarily include qPCR, MFC, and NGS. A broad consensus exists regarding the limitations of these techniques in fully characterizing MRD and in using MRD to establish diagnosis or prognosis [77,104,105]. qPCR is a well-standardized technique with high sensitivity, offering high sample throughput and rapid turnaround times; however, this advantage applies only to patients with specific genomic abnormalities. Indeed, qPCR is applicable to only a subset of patients because it relies on the presence of defined mutations or translocations, and does not adequately capture clonal heterogeneity or evolution. Moreover, qPCR provides only relative quantification of targets within the analyzed sample, typically by comparison with a standardized transcript, such as ABL1. In contrast, MFC has broader applicability and can be used in nearly all patients, including approximately half of patients with AML who lack a specific mutation or fusion gene detectable by qPCR.

However, the sensitivity is lower than that of PCR, and MFC is difficult to standardize because of differences in laboratory equipment, sample processing, and cytometer configuration, leading to poor comparability between institutions. This technique utilizes gene panels to detect molecular aberrations and allows in-depth analysis of a large number of genes. However, a major challenge is the inability to distinguish true AML-specific mutations from those associated with clonal hematopoiesis. Mutations in genes such as DNA methyltransferase 3 alpha, Tet methylcytosine dioxygenase 2, or additional sex combs like 1 are common in age-related clonal hematopoiesis and should not be considered MRD in isolation. Currently, NGS is not recommended as a single MRD detection method.

Newer exploratory techniques, including dPCR, combinatorial approaches, and liquid biopsies, are being investigated to overcome some of these limitations. However, dPCR shows high variability depending on the sample material (peripheral blood vs. BM) and may not cover all types of mutations [77]. Despite their strong prognostic value, key challenges include variable ctDNA shedding (especially in small, indolent, or sanctuary-site diseases), assay standardization, false-negative and false-positive results, and the definition of actionability thresholds across tumor types. Large randomized trials are needed to demonstrate that altering therapy solely based on ctDNA MRD status improves survival; therefore, most applications remain confined to clinical trials or specialized centers rather than being universally incorporated into treatment guidelines [46,106].

Although MRD detection is a valuable and increasingly important tool in oncology, its limitations, such as variability in sensitivity, lack of standardization, and the need for careful interpretation, must be considered when it is used for clinical prognosis and diagnosis. Therefore, MRD assessments should not replace comprehensive clinical evaluations; rather, they complement other diagnostic and prognostic tools needed to guide therapy [33]. Furthermore, ongoing efforts address regulatory considerations from the FDA and industry to validate MRD as a surrogate endpoint to expedite drug development, highlighting the need for greater collaboration and standardized trial designs. The push to standardize and implement MRD assessment is not an isolated effort, but a major collaborative initiative [36].

## 7. Conclusions and Future Directions

MRD assessment has become a transformative and indispensable tool in oncology, defined by the presence of a very small number of cancer cells that remain after curative treatment and are undetectable by conventional diagnostic methods. MRD assessment provides powerful prognostic and predictive insights into both hematological malignancies and solid tumors.

In hematologic malignancies such as ALL and AML, the utility of MRD is well established; MRD negativity is consistently and strongly associated with improved PFS and OS, whereas MRD positivity is uniformly linked to higher relapse risk and inferior survival outcomes. The clinical utility of MRD in these diseases is recognized by major regulatory bodies, with the EMA supporting MRD as a surrogate efficacy endpoint in clinical studies.

For solid tumors, the application of MRD has advanced significantly, primarily through liquid biopsy technologies that detect ctDNA. ctDNA-based MRD monitoring provides dynamic, non-invasive surveillance, enabling the prediction of recurrence months before it becomes detectable by imaging in cancers such as CRC, NSCLC, and breast cancer. Accurate detection relies on highly sensitive techniques, including flow cytometry, qPCR, NGS, and dPCR. Among these, NGS has emerged as a powerful tool, offering broad applicability and the ability to detect very low levels of residual disease. The future of MRD monitoring is moving toward high-resolution diagnostics through the integration of multi-omics (such as transcriptomics and proteomics) and single-cell sequencing analyses, which enhance diagnostic sensitivity, characterize residual disease, and support the development of truly personalized cancer care.

The paradigm of cancer care is undergoing a fundamental transition from a cure-oriented approach to a long-term management strategy, encompassing early diagnosis, post-treatment surveillance, and recurrence monitoring. Within this framework, MRD has emerged as a critical component of precision oncology and serves as a sensitive indicator of treatment responses and disease dynamics. Advances in medical technologies, including early cancer detection and the development of high-performance anticancer therapeutics, have underscored the clinical importance of MRD assessments, particularly in aging societies. Notably, key MRD detection technologies are directly applicable to early cancer detection, highlighting the urgent need for research to improve analytical sensitivity and specificity. As clinical and technological datasets related to MRD continue to accumulate, the integration of AI-based approaches is expected to further enhance MRD applications, ultimately enabling more cost-effective, efficient, and patient-centered cancer management strategies.

Several challenges must be addressed before MRD-guided management can be universally implemented. These include assay standardization, biological variability in ctDNA shedding, interpretation of low-level positivity, and the establishment of clinically actionable thresholds across tumor types [37,95,96,104,105]. Notably, large prospective trials are required to demonstrate that modifying therapy based on the MRD status translates into meaningful improvements in survival, particularly in solid tumors.

In summary, MRD serves as a high-resolution molecular lens through which clinicians can detect, monitor, and intercept cancer recurrence. As technological, analytical, and clinical frameworks continue to mature, the integration of patient-informed MRD with advanced computational and multi-omics platforms is expected to redefine precision oncology, shifting the focus from static endpoints of cure to dynamic personalized disease management.

## Data Availability

Data sharing is not applicable to this article. No new data were created or analyzed in this study.

## Abbreviations

The following abbreviations are used in this manuscript:

ABL1	Abelson tyrosine-protein kinase 1
ADHFE1	alcohol dehydrogenase iron containing 1
AI	Artificial intelligence
ALL	acute lymphoblastic leukemia
alloSCT	allogeneic stem cell transplantation
AML	acute myeloid leukemia
ASCT	autologous stem cell transplantation
BCL2	B cell lymphoma2
BM	bone marrow
cfDNA	cell-free DNA
CLL	chronic lymphocytic leukemia
CML	chronic myeloid leukemia
CRC	colorectal cancer
CTC	circulating tumor cell
ctDNA	circulating tumor DNA
ddPCR	droplet-based dPCR
dPCR	digital PCR
DTC	disseminated tumor cell
EDGE	Enrichment and detection using Genomic Enrichment
EGFR	epidermal growth factor receptor
ELN	European LeukemiaNet
EMA	European Medicines Agency
EMT	epithelial–mesenchymal transition
ER	estrogen receptor
EV	tumor-derived extracellular vesicle
FLT3	fms-like tyrosine kinase 3
GC	gastric cancer
GMM	Gaussian mixture model
GPRC5D	G protein-coupled receptor, class C, group 5, member D
HER2	human epidermal growth factor receptor
IDH	isocitrate dehydrogenase
ITD	internal tandem duplication
MFC	multiparameter flow cytometry
MM	multiple myeloma
MRD	minimal residual disease
NGS	next-generation sequencing
NPM1	Nucleophosmin
NSCLC	non-small cell lung cancer
OS	overall survival
PCR	polymerase chain reaction
PFS	progression-free survival
Ph	Philadelphia
Ph-positive ALL	Philadelphia chromosome-positive Acute Lymphoblastic Leukemia
PIK3CA	phosphatidylinositol 3-kinase catalytic subunit alpha
PML	promyelocytic leukemia
PPP2R5C	protein phosphatase 2 regulatory subunit B gamma
qPCR	quantitative real-time PCR
RFS	recurrence-free survival
RUNX1T1	RUNX family transcription factor 1 partner transcriptional co-repressor 1
scMRD	single-cell MRD
scRNA-seq	Single-cell RNA sequencing
SDC2	syndecan2
TFR	treatment-free remission
TP53	tumor protein p53
TSG	tumor suppressor gene
UDS	ultra-deep sequencing
WGS	whole-genome sequencing
